# An Enhanced Bounding Surface Model for Modelling Various Cyclic Behaviour of Clay

**DOI:** 10.3390/ma15217609

**Published:** 2022-10-29

**Authors:** Junxiang Wang, Giovanna Xotta, Nico De Marchi, Valentina Salomoni

**Affiliations:** 1 Department of Civil, Environmental and Architectural Engineering, University of Padua, 35131 Padova, Italy; 2 Department of Management and Engineering, University of Padua, 36100 Vicenza, Italy

**Keywords:** clays, cyclic loading, hysteresis loops, bounding surface plasticity, hardening modulus, damage factor

## Abstract

Many results from cyclic triaxial experiments indicate that porous media, such as clays, exhibit various long-term behaviours under different cyclic stress ratios (CSRs). These can be classified into three main categories, namely, cyclic shakedown, cyclic stable and cyclic failure. Modelling these soil deformation responses, along with pore pressure and other fundamental cyclic aspects, such as closed hysteresis cycles and degradation, is still an open challenge, and research to date is limited. In order to properly describe and capture these characteristics, an enhanced plasticity model, based on the bounding surface and stress distance concepts, is developed here. In detail, a new uniform interpolation function of the plastic modulus, suitable for all loading stages, is proposed, and a new damage factor associated with the plastic shear strain and the deformation type parameter, is also incorporated into the plastic modulus. Accordingly, cyclic shakedown and cyclic failure can be distinguished, and degradation is achieved. Closed hysteresis loops, typical of clays, are obtained through a radial mapping rule along with a moving projection centre, located by the stress reversal points. Comparisons between the obtained numerical results and the experimental ones from literature confirm the suitability of the constitutive approach, which is capable of correctly capturing and reproducing the key aspects of clays’ cyclic behaviour.

## 1. Introduction

Civil structures such as marine wind towers, embedded offshore structures and highway foundations are often exposed to cyclic loads from wind, ocean waves and traffic loads. Therefore, evaluating the cyclic response of the soil foundation is of relevance, as it influences the long-term behaviour of the supporting structures.

**Figure 1:**
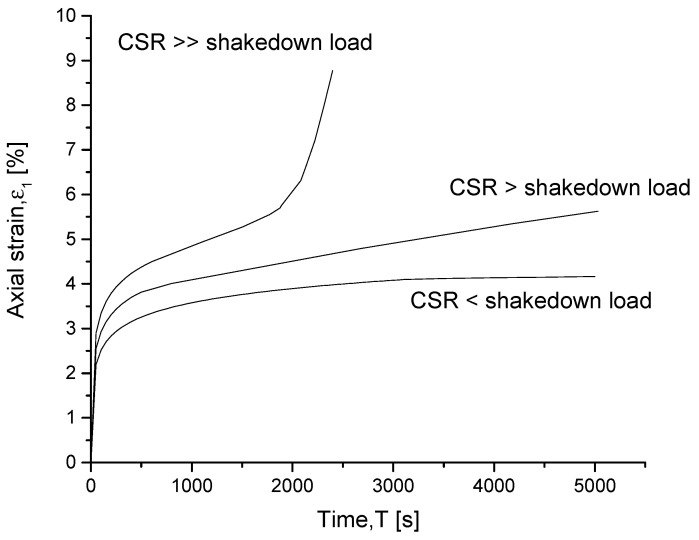
Influence of CSR on clay cyclic stress–strain behaviour [6].

Cyclic triaxial tests conducted on clays have shown that cyclic deformation of soil is characterised by elasto-plastic unloading, closed hysteresis loops and strain accumulation [1,2,3,4,5]. In detail, when the repeated load or cyclic stress ratio (CSR) is lower than a threshold value (“shakedown load”), clay, after several cyclic loads, will reach the shakedown state, exhibiting purely elastic behaviour and no longer producing any accumulation of plastic deformation. On the other hand, when the repeated load is significantly higher than the shakedown load, soil will produce more and more plastic strain with each cyclic load and, after several cycles, it will finally fail. Another case is when the repeated load is slightly higher than the threshold value load; in this case, the soil will produce plastic strain at a constant rate along with each cyclic load, and the permanent strain will grow at a constant rate as well. These features are depicted in Figure 1, for different CSRs. These characteristic deformation aspects are rarely modeled and should be considered as essential elements whenever constitutive models, for the cyclic behaviour of soil, are developed.

Compared to monotonic loading, soil deformation behaviour under cyclic loading is more complex, and it has attracted the interest of many researchers. Gu et al. [6] investigated the one-way cyclic behaviour of remoulded saturated clay samples via a true triaxial apparatus, which allows control over the CSR, the cyclic intermediate principal stress and the over-consolidation ratio (OCR) factors. Guo et al. [7] performed a series of cyclic triaxial tests to analyze the long-term cyclic behaviour of saturated soft clays considering different drainage conditions. Chiaro et al. [8] studied the undrained cyclic deformation behaviour of loose saturated sands under different static shear stresses. Kong et al. [9] conducted cyclic triaxial tests on Zipingpu gravel and successfully employed the discrete element method to study the stress–dilatancy relationship of gravelly soils, whereas Sze and Yang [10] investigated the influence of the specimen preparation on the deformation behaviour of saturated sands under cyclic loading. Zhuang et al. [11] studied the effects of the initial deviatoric stress state and principal stress orientation on the strain components and shear strength of soft marine clay. Yang et al. [12] reported a comprehensive experimental investigation of the undrained cyclic behaviour of saturated loose sand with static shear, under triaxial compression and extension conditions. Similarly, Wang et al. [13] conducted dynamic triaxial tests, with different initial static shear stresses and overconsolidated ratios, to study the cyclic behaviour of marine clay.

However, despite the amount of experiments performed, research on constitutive modelling of soil cyclic behaviour is relatively limited.

Conventional elastoplastic geomechanical models, such as the Cam Clay (CC) [14] model, fail to capture the typical behaviour described above, as it is not possible to consider the reversed plastic deformations from a single expanding yield surface. To tackle this difficulty, Mroz [15,16,17] proposed the idea of a multi-surface along with kinematic hardening model: an outer surface, corresponding to the maximum loading stress, together with a group of overlapping but disjointed and geometrically similar yield surfaces, are defined to model the plastic deformation by moving the yield surfaces within the outer surface during the entire loading process. Although the multi-surface models can capture the cyclic behaviour of soil, they require a considerable computational effort and result in discontinuous stress–strain curves due to the abrupt change when a stress point crosses two different surfaces.

To overcome the complexity of models with multiple yield surfaces, Dafalias [18,19] proposed the bounding surface model that defines only the initial loading surface and the bounding surface. The function of overlapping yield surfaces is replaced by a suitable interpolating function between the stress point and a image stress point on the bounding surface. Another difference between the bounding surface model and the classical elastoplastic and multisurface models lies in the fact that the plastic modulus *h* also relies on the distance between the stress point and the bounding surface, thus ensuring a smooth transition from the elastic to the plastic condition and reducing the computational efforts. Additionally, Dafalias and Hemnann [20] degenerated the loading surface into a loading point, and the two-surfaces model was transformed into a single-surface model. Recently, Seidalinov [21] extended the well-known SANICLAY [22] model by employing the concept of bounding surface for modelling the response of clay under cyclic loading. Zhao [23] also incorporated salt contents and confining pressures into this anisotropic bounding surface model to study the dynamic characteristics of frozen saline soils. Zymnis [24] adopted the Tsinghua ThermoSoil model [25] to study the thermo-mechanical response of clays under long-term heating and cooling cycles in fully drained conditions. Chen [26] took the stress distance concept and presented a two-surface plasticity model to describe some important characteristics of saturated clay under cyclic loading, such as closed hysteresis loops, cyclic shakedown and degradation. Li [27] developed a new type of anisotropic kinematic hardening rule of the loading surface along with the stress distance concept, to predict the undrained behaviour of saturated cohesive soils subjected to cyclic load. The above, and many other works, such as [28,29,30,31], confirm how the idea of stress distance and bounding surface is and has been widely used to model the behaviour of soils under cyclic loading conditions.

Instead of developing a constitutive model exclusively for clay, Yu [32,33] proposed a unified bounding surface model to simulate the cyclic behaviour of both clay and sand, and developed three different hardening modulus interpolation functions for the virgin loading, unloading and reloading processes, respectively. The accumulated plastic strain was correctly captured, but not the closed hysteresis, since the projection center remains the same for the unloading and reloading stages. Khalili and Kan [34,35,36] adopted the same form of bounding surface and introduced the second segment of CSL to account for the particle crushing effect of granular materials under a high level of shear stress; using the two different CSL slopes, the model’s ability to predict the deformation behaviour of sand under large shear stresses is greatly improved. In addition, Zhou [37] successfully used the same bounding surface shape to model the thermomechanical behaviour of clays under small and large strains and Ma [38] incorporated rotational and distortional hardening rules to model the monotonic behaviour of anisotropic consolidated clays. Moghadam [39] adopted a similar yield/bounding surface and developed a new dilatancy relation to simulate, in a unified framework, the monotonic and cyclic behaviour of clays and sands.

Although the aforementioned models can satisfactorily predict the initial or short-term cyclic behaviour of soils, they ignore a fundamental characteristic of cyclic loading on soil foundations; namely that repeated loading, such as ocean wave or transportation loading, should last for a long period. The long-term loading condition would, in the end, result in the three deformation characters described previously. Thus, the capability to model these behaviours is needed when dealing with the long-term cyclic behaviour of soils and to date, due to the limited research on this aspect, this is still a challenge.

In the present paper, the cyclic behaviour of soil is thus classified into three categories: shakedown, stable, and failure. A similar yield surface, such as that of the CASM [33], is adopted to investigate these three types of clay deformation behaviour. Specifically, a novel uniform interpolation function of the plastic modulus is proposed, which is suitable for all the loading phases and, to distinguish cyclic shakedown and cyclic failure, a new damage factor associated with the plastic shear strain and the deformation type parameter is incorporated into the model. Closed hysteresis loops are here achieved through the adoption of a radial mapping rule which passes through stress reversal points, allowing us to model the non-linear stress–strain behaviour under unloading and reloading. The model is finally validated against several triaxial cyclic tests taken from literature, thus confirming its ability to satisfactorily simulate the deformation behaviour of clays under cyclic loading.

This paper is organised as follows. Section 2 describes the proposed constitutive model. Special attention is paid to the newly introduced hardening modulus function and to the method for modelling the three different cyclic behaviours of clay. Section 3 presents the implementation of the model in the commercial FEM software ABAQUS. The two-step explicit integration with the auto-error control method is given in detail. Section 4 validates the model against undrained cyclic triaxial tests from the literature and confirms its ability to successfully capture the different cyclic responses. Finally, Section 5 gives the conclusions and future research perspectives.

## 2. Constitutive Model

### 2.1. Incremental Relations

Small strains and additive decomposition are assumed, hence

(1) $$\dot{\varepsilon }={\dot{\varepsilon }}^{e}+{\dot{\varepsilon }}^{p}$$

and stress rate within the elastic field is

(2) $${\dot{\sigma }}^{\prime }=D^{e}:\dot{\varepsilon^{e}}$$

where $D^{e}$ is the elasticity tensor and defined by the bulk modulus *K* and the shear modulus *G*:

(3) $$\begin{array}{cc} K & =\frac{\left(1+e\right)}{\kappa }p^{\prime }=\frac{vp^{\prime }}{\kappa }\\ G & =\frac{3(1-2\mu )K}{2(1+\mu )}=\frac{3(1-2\mu )}{2(1+\mu )}\frac{vp^{\prime }}{\kappa } \end{array}$$

where μ is the Poisson’s ratio, v=(1+e) is the specific volume, and κ is the slope of the loading–unloading line in the $e-\ln p^{\prime }$ plane.

The plastic strain rate is defined as

(4) $${\dot{\varepsilon }}^{p}=\frac{1}{h}m⊗\tilde{n}:{\dot{\sigma }}^{\prime }$$

where $\tilde{n}$ and m are the unit normal vectors to the bounding surface and plastic potential at $\sigma^{\prime }$, respectively, and *h* is the hardening modulus.

By combining Equations (1), (2) and (4), the following incremental elasto-plastic stress–strain relationship can be obtained:

(5) $${\dot{\sigma }}^{\prime }=D^{ep}:\dot{\varepsilon }=\left(D^{e}-\frac{D^{e}:m⊗\tilde{n}:D^{e}}{h+\tilde{n}:D^{e}:m}\right):\dot{\varepsilon }$$

### 2.2. Bounding Surface Plasticity

The main components of the proposed model are briefly recalled here:

1. a bounding surface, that serves as the outer bound of the stress state in the stress space;
2. a linear mapping rule with a moving projection centre locating the image stress on the bounding surface;
3. a plastic potential defining the direction of the plastic flow;
4. a uniform interpolation function of the plastic modulus for all the loading phases;
5. a damage factor, associated with the plastic shear stress, incorporated into the plastic modulus to obtain a degradation to distinguish between cyclic shakedown and cyclic failure.

Following previous studies [40,41], the model adopts the critical state theory to define the ultimate condition that the soil reaches with an increasing deviatoric shear strain. In the $p^{\prime }-q$ plane, with $p^{\prime }$ and *q* being the mean and deviatoric stresses, respectively, the slope of the critical state line (CSL) can be expressed as a function of the drained friction angle $\phi^{\prime }$ as:

(6) $$M_{cs}={\left(\frac{q}{p^{\prime }}\right)}_{cs}=\frac{6\sin\phi^{\prime }}{3-\sin\phi^{\prime }}$$

In the $e-\ln p^{\prime }$ plane, where *e* is the void ratio, the CSL is parallel to the isotropic compression line (ICL) and it can be defined by the critical void ratio, $e_{cs}$, as:

(7) $$e_{cs}=\Gamma_{o}-\lambda \ln\left(p^{\prime }\right)=\Gamma_{o}-\lambda \ln\left(p_{c}^{\prime }/r\right)$$

where: $\Gamma_{o}$ is specific volume at $p^{\prime }=1$, λ is the ICL slope, $p_{c}^{\prime }$ is the preconsolidation pressure and *r* is the spatial ratio that determines the vertical distance between the CSL and the normal consolidation line (NCL) in the $e-\ln p^{\prime }$ plane [32].

The bounding surface can be expressed, in the $p^{\prime }-q$ space, as:

(8) $$F={\left(\frac{\bar{q}}{M_{cs}{\bar{p}}^{\prime }}\right)}^{n}+\frac{\ln({\bar{p}}^{\prime }/{\bar{p}}_{c})}{\ln r}$$

The image stress point (${\bar{p}}^{\prime },\bar{q}$) is obtained by an appropriate mapping rule projecting the true stress point ($p^{\prime },q$) onto the bounding surface. The spacing ratio *r*, introduced in Equation (7), defines in the $p^{\prime }-q$ plane the intersection point of the critical state line with the bounding surface [32]. The model parameter *n* defines the curvature of the surface, while the hardening internal variable ${\bar{p}}_{c}$ controls its size and is a function of the volumetric plastic strain.

**Figure 2:**
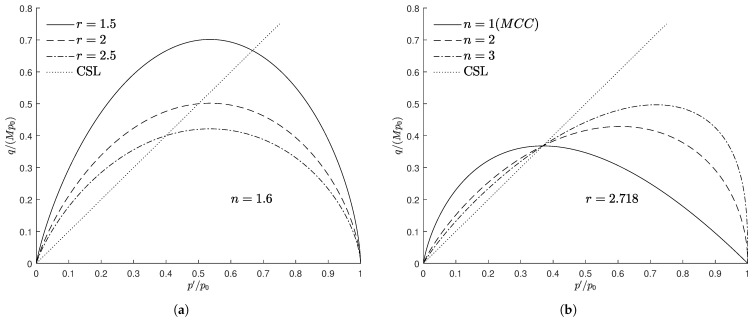
Influence of (**a**) parameter *r* and (**b**) parameter *n* on the bounding surface.

Equation (8) has been extensively used in previous works to define the bounding surface, since it allows different forms of bounding surface to be obtained through a single formulation. For example, the original CC surface is fully retrieved by choosing n=1 and r=2.718 while, when n=1.6 and r=2, the wet side of the Modified Cam Clay (MCC) model can also be accurately matched by the bounding surface, as shown in Figure 2. For details about the influence of *n* and *r* on the bounding surface, readers are referred to Zhou [37].

A non-associated flow rule is assumed here and the plastic potential, in agreement with [34], is expressed as

(9) $$g=\bar{q}+M_{cs}{\bar{p}}^{\prime }\ln\left(\frac{{\bar{p}}^{\prime }}{{\bar{p}}_{c}}\right)$$

### 2.3. Mapping Rule

In the context of bounding surface plasticity, plastic strains occur when $\sigma^{\prime }$ moves within the bounding surface or lies on it. This is accomplished by defining the hardening modulus *h* as a decreasing function of the distance between the true and the ‘image’ stress points, $\sigma^{\prime }$ and ${\bar{\sigma }}^{\prime }$, on the bounding surface.

In the traditional bounding surface model, ${\bar{\sigma }}^{\prime }$ is obtained through a radial mapping rule in which the coordinate origin or the initial stress point is adopted as the projection center and is kept unchanged during all loading phases. This mapping rule is successfully applied under monotonic loading conditions, but it is unable to deal with the typical hysteresis or soil degradation phenomena under cyclic loading conditions. The reason is that either unloading is treated as elastic, or the hardening modulus cannot capture the plastic behaviour of the soil once the stress reversal occurs.

**Figure 3:**
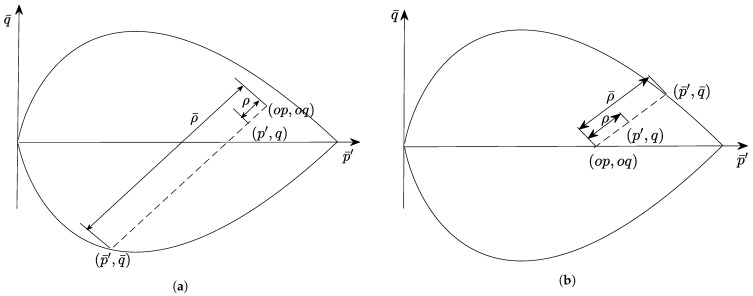
Mapping rule and projection centre at different loading stages: (**a**) unloading and (**b**) reloading.

According to Masing’s rule, unloading can be considered as a reverse loading process corresponding to the previous loading, and thus the reverse stress point can be regarded as the starting point of the reverse loading. Therefore, here, it is assumed that the projection centre immediately moves to the stress reverse point when the loading direction changes. More precisely, during the unloading phase the last point of the previous loading is taken as the mapping origin, as shown in Figure 3a, while for the subsequent reloading, the mapping origin immediately moves to the starting point of this phase, as shown in Figure 3b.

The stress reverse point is identified by the loading index L≤0, calculated by imposing the consistency condition on the bounding surface as

(10) $$L=[\left(\partial F/\partial {\bar{p}}^{\prime }\right)\;{\dot{p}}^{\prime }+\left(\partial F/\partial \bar{q}\right)\;\dot{q}]/h$$

A linear mapping rule is adopted to locate the image stress

(11) $$\begin{array}{c} {\bar{p}}^{\prime }=\frac{\bar{\rho }}{\rho }\left(p^{\prime }-op\right)+op \end{array}$$

(12) $$\begin{array}{c} \bar{q}=\frac{\bar{\rho }}{\rho }\left(q-oq\right)+oq \end{array}$$

where $\bar{\rho }$ is the Euclidean distance between (op,oq) and (${\bar{p}}^{\prime },\bar{q}$) and ρ between (op,oq) and ($p^{\prime },q$).

### 2.4. Plastic Modulus

In the context of bounding surface plasticity, the plastic modulus *h* at the actual stress point $\left(p^{\prime },q\right)$ is related to that, $h_{b}$, at the image stress point $\left({\bar{p}}^{\prime },\bar{q}\right)$ by the Euclidean distance between these two points. Therefore, the plastic state is not restricted only to that lying on F=0, as in classical plasticity, and this is the major benefit of the bounding surface concept.

The hardening modulus is here defined as

(13) $$\begin{array}{c} h=h_{b}+h_{f} \end{array}$$

where $h_{b}$ is the hardening modulus of ${\bar{\sigma }}^{\prime }$ on the bounding surface and $h_{f}$ is a function of the distance from the stress point to the bounding surface. This is a new damage state variable *d* that will be discussed later.

The volumetric plastic strain rate, ${\dot{\varepsilon }}_{v}^{p}$, can be expressed through the flow rule

(14) $$\begin{array}{c} {\dot{\varepsilon }}_{v}^{p}=\dot{\Lambda }\;m_{p} \end{array}$$

(15) $$\begin{array}{c} m_{p}=\left(\partial g/\partial {\bar{p}}^{\prime }\right)/∥\partial g/\partial {\bar{\sigma }}^{\prime }∥ \end{array}$$

where Λ is the plastic multiplier.

By forcing the image stress to remain on the bounding surface, the consistency condition can be expressed as

(16) $$\dot{F}=\left(\frac{\partial F}{\partial \bar{\sigma^{\prime }}}\right):{\dot{\bar{\sigma }}}^{\prime }+\left(\frac{\partial F}{\partial {\bar{p}}_{c}}\frac{\partial {\bar{p}}_{c}}{\partial \varepsilon_{v}^{p}}\right)\;{\dot{\varepsilon }}_{v}^{p}=0$$

By replacing the derivative of the bounding surface with the vector

(17) $$\tilde{n}=\left(\partial F/\partial {\bar{\sigma }}^{\prime }\right)/∥\partial F/\partial {\bar{\sigma }}^{\prime }∥$$

and introducing Equation (14), Equation (16) becomes

(18) $$\tilde{n}:\dot{{\bar{\sigma }}^{\prime }}-\dot{\Lambda }\;h_{b}=0$$

in which $h_{b}$ is equal to

(19) $$h_{b}=-\frac{\partial F}{\partial {\bar{p}}_{c}}\frac{\partial {\bar{p}}_{c}}{\partial \varepsilon_{v}^{p}}\frac{m_{p}}{∥\partial F/\partial {\bar{\sigma }}^{\prime }∥}$$

with

(20) $$\frac{\partial F}{\partial {\bar{p}}_{c}}=-\frac{1}{{\bar{p}}_{c}\ln\left(r\right)}$$

The bounding surface undergoes isotropic hardening when the image stress moves on the surface; in this case, the hardening law takes the form:

(21) $$d{\bar{p}}_{c}=\frac{v{\bar{p}}_{c}}{\left(\lambda -\kappa \right)}d\varepsilon_{v}^{p}$$

Therefore, by substituting Equations (15), (20), (21) into Equation (19), the hardening modulus at the bounding surface can be written as:

(22) $$h_{b}=\frac{v}{\left(\lambda -\kappa )\ln(r\right)}\frac{m_{p}}{||\partial F/\partial {\bar{\sigma }}^{\prime }\left|\right|}$$

Here, a novel $h_{f}$ is proposed, such that it results equal to zero on the bounding surface and infinite when stress reversion occurs

(23) $$\begin{array}{c} h_{f}=\frac{v}{\left(\lambda -\kappa )\ln(r\right)}cot\left(\frac{\pi }{2}\gamma \right){k_{f}}^{t} \end{array}$$

where $\gamma =\rho /\bar{\rho }$ is the distance ratio and the deformation type parameter *t* is used to distinguish between cyclic shakedown (t<0), cyclic failure (t>0) and cyclic stable (t=0) character. The detailed explanation of how *t* distinguishes these three phases is discussed below.

$k_{f}$ is a new hardening parameter controlling the plastic modulus in order to properly simulate the continuous evolution of the closed hysteresis curve and the degradation during cyclic loading, proposed as a decaying function of the deviatoric plastic strain, $\varepsilon_{d}^{p}$

(24) $$\begin{array}{c} k_{f}=\left[1-k_{1}\;\left(1-\exp\left(d\right)\right)\right]k_{0} \end{array}$$

(25) $$\begin{array}{c} \dot{d}=k_{2}\;\left|{\dot{\varepsilon }}_{d}^{p}\right| \end{array}$$

where $k_{0}$ is the initial value of $k_{f}$ and $k_{1}$, $k_{2}$, *d* are new model state variables that can assume different values for the reloading and unloading processes, in order to better model the cyclic behaviour of soil. Furthermore, from Equation (25) it can be noted that the damage factor *d* remains either unchanged or increases linearly during the loading process with the module of $\varepsilon_{d}^{p}$.

**Figure 4:**
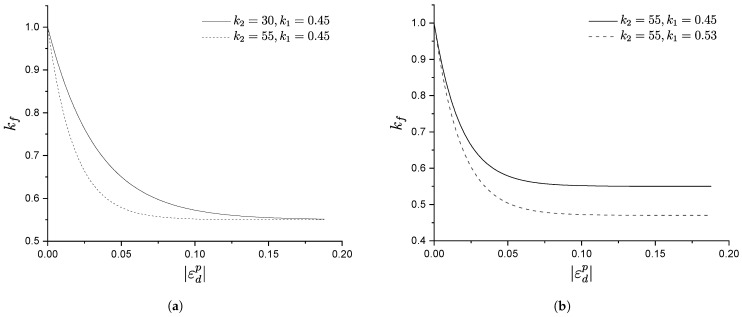
Influence of $k_{1},k_{2}$ on $k_{f}$. (**a**) Influence of $k_{2}$ on $k_{f}$, (**b**) Influence of $k_{1}$ on $k_{f}$.

Figure 4 illustrates the evolution of $k_{f}$ along with $|\varepsilon_{d}^{p}|$. It can be observed that, with the accumulation of the absolute deviatoric plastic strain, the hardening parameter $k_{f}$ decreases, and in turn leads to decreasing $h_{f}$ and the achievement of stiffness degradation. Additionally, it can also be noticed that parameter $k_{2}$ controls the decrease rate of $k_{f}$, while $k_{1}$ controls the decrease magnitude.

**Figure 5:**
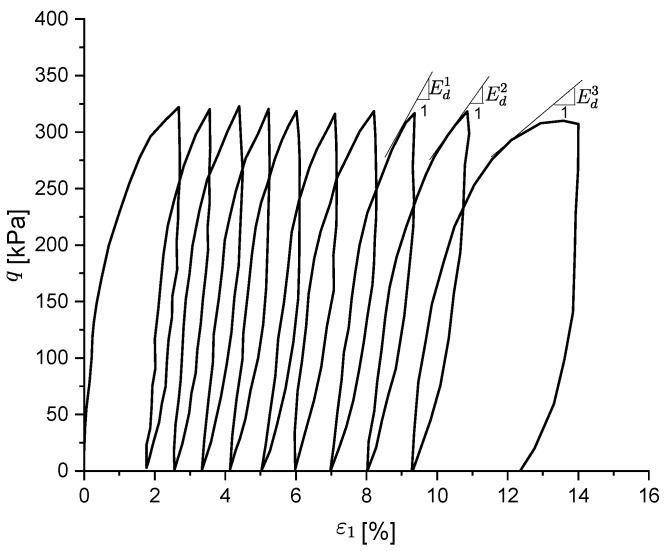
Cyclic failure under a high CSR.

When the CSR is much higher than the shakedown load, the soil will tend to fail after a certain number of loading cycles, as shown in Figure 5. In detail, it can be seen that the permanent deformation accumulated at each loading cycle increases significantly after the first several cyclic loads; this is due to the evident stiffness deterioration ($E_{d}^{1}>E_{d}^{2}>E_{d}^{3}$) that the soil presents at each subsequent reloading phase. In order to model such behaviour under a high CSR, the parameter *t* is introduced to control the stiffness degradation at reloading.

**Figure 6:**
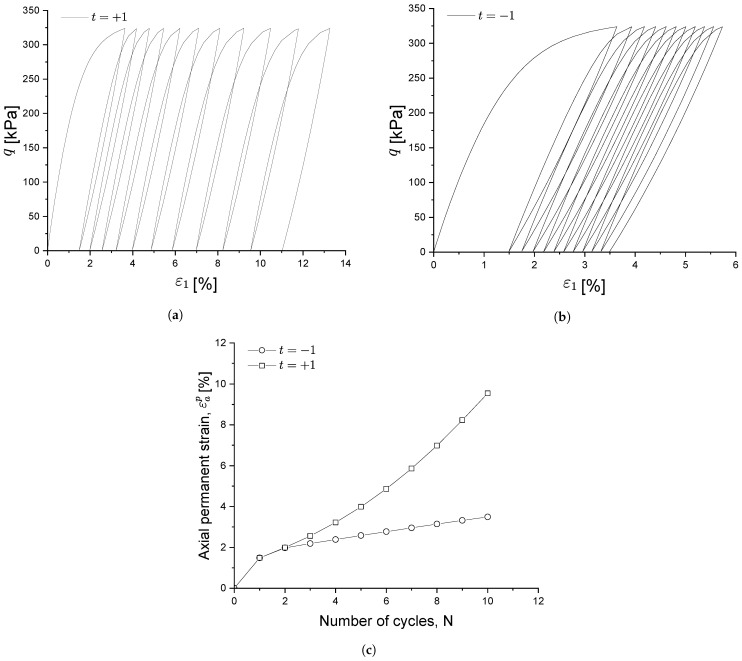
Influence of parameter *t*. (**a**) t=+1, (**b**) t=−1, (**c**) Deformation velocity at variable *t*.

Figure 6 illustrates the influence of the sign of parameter *t* on the deformation properties of the soil subjected to cyclic loading. Particularly, in Figure 6c, it can be appreciated that the accumulation strain increases rapidly as the number of loading cycles increases when the sign of *t* is positive, while it increases at a stable rate when the sign of *t* is negative.

Parameter *t* works in such a way that, since $k_{f}$ is a decaying function along with the development of the plastic shear strain (see Equation (24)), when the sign of *t* is positive during the reloading stage, the plastic modulus will also decrease and leads to stiffness degradation. Therefore, the soil exhibits more plastic behaviour and the corresponding permanent strain at each loading cycle increases, as shown in Figure 6a,b.

**Figure 7:**
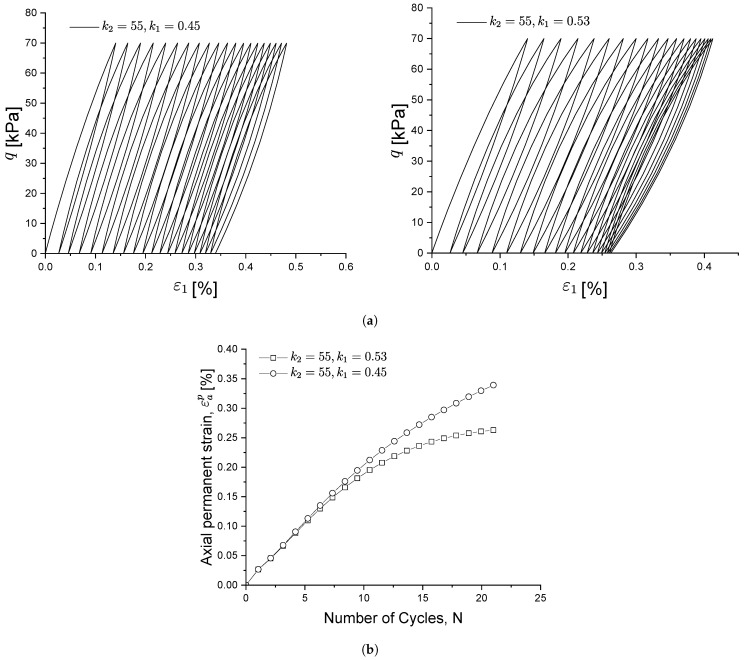
Influence of parameter $k_{1}$ on the cyclic stress-strain response (**a**) and on the permanent soil strain under cyclic loading (**b**).

Figure 7 shows the influence of $k_{1}$ on the soil cyclic behaviour and strain accumulation. In particular, from Figure 7a, it can be observed that, for higher values of $k_{1}$, the soil reaches the shakedown state faster and smaller permanent strains are produced as it is recovered during the unloading phase, as depicted in Figure 7b. From Figure 4 and Equation (24), it can be observed that $k_{f}$ is smaller for larger values of $k_{1}$, which in turn leads to a smaller plastic modulus and results in stiffness degradation. Thus $k_{1}$ plays the role of controlling whether or not the soil will reach the shakedown state.

**Figure 8:**
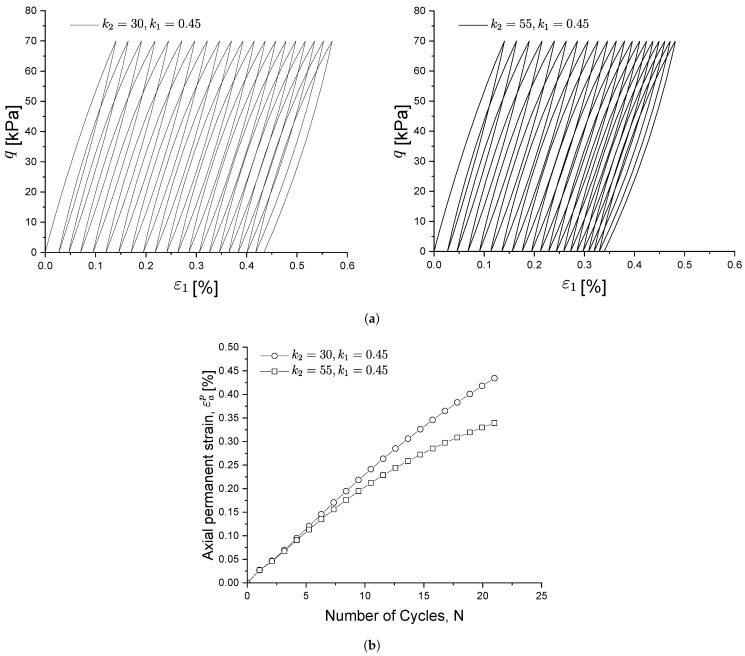
Influence of parameter $k_{2}$ on the cyclic stress-strain relationship (**a**) and on the permanent strain under cyclic loading (**b**).

Similarly, Figure 8 shows the influence of $k_{2}$ on soil behaviour: at a CSR slightly higher than the shakedown load, the soil will not reach a shakedown state and will continuously produce plastic strains at each load cycle. Parameter $k_{2}$ is therefore used to control the evolution of the cyclic stress–strain behaviour under the above conditions. With smaller $k_{2}$ values, the closed hysteresis curve moves forward faster, as shown in Figure 8a. As a result, $k_{f}$ will decrease more slowly (see Figure 4 and Equation (24)) and so will the plastic modulus, which results in a slower decrease in the stiffness degradation and less strain recovery during the unloading stage. This is also evident in Figure 8b, where the development of accumulated strain increases more quickly with a smaller $k_{2}$.

**Figure 9:**
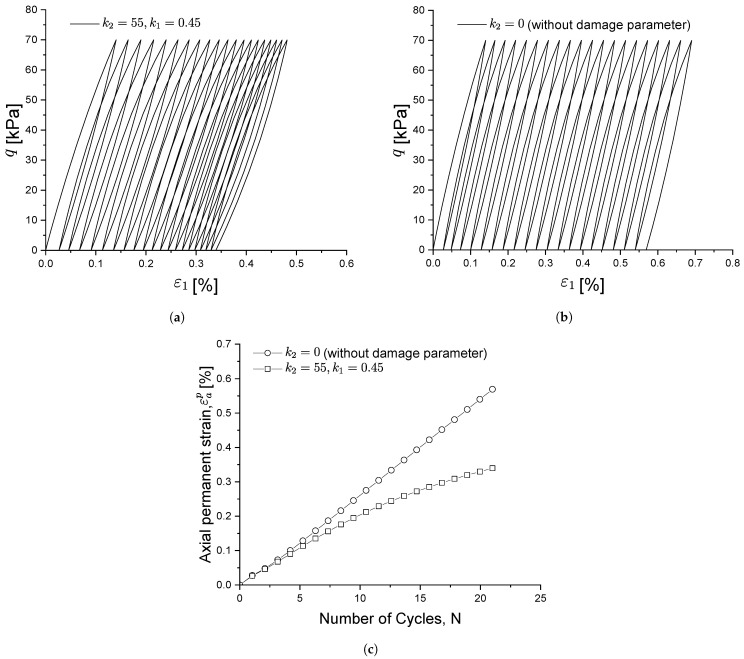
Influence of damage factor *d*. (**a**) with damage factor, (**b**) without damage factor, (**c**) influence of damage factor on the permanent soil strain under cyclic loading.

Figure 9 shows the influence of the newly introduced damage factor on the cyclic stress–strain behaviour. It can be seen that, when there is no damage factor (t=0), the accumulation strain increases linearly with load cycles.

With the introduction of the damage factor *d*, and of $h_{f}$, the capability of the model is improved, and it is made capable of simulating the hysteresis curve well for different cyclic behaviours. Unlike other bounding surface constitutive models [23,32], which treat the initial, unloading, and reloading phases separately—which results in a different form of $h_{f}$ for each loading stage—the $h_{f}$ proposed here has a uniform equation (see Equation (23)) for the entire cyclic process.

### 2.5. Model Parameters

Two sets of constitutive parameters are employed for the updated bounding surface model proposed here: the isotropic parameters ($\kappa ,\mu ,\lambda ,M_{cs},{\bar{p}}_{c},r,n$) and the cyclic plasticity $(k_{0},k_{1},k_{2},t$) parameters.

In detail, and along with the considerations for the newly introduced cyclic plasticity parameters given in the previous section:

1. The isotropic bounding surface parameters $\kappa ,\mu ,\lambda ,M_{cs}$ and ${\bar{p}}_{c}$ are the traditional parameters used in the MCC model. The elastic swelling slope, κ, and Poisson’s ratio, μ, used to model the elastic behaviour, can be obtained from isotropic compression tests. λ is the slope of the normal consolidation line, in the $e-\ln p^{\prime }$ plane, and $M_{cs}$ is the critical stress ratio. They can be calibrated by drained or undrained triaxial tests. ${\bar{p}}_{c}$ is the preconsolidation pressure, which defines the initial size of the bounding surface.
2. *n* and *r* define the shape of the bounding surface. They can be better obtained by adjusting the bounding surface to the response of the material under undrained conditions during the initial loading.
3. *t* is the deformation type parameter used to distinguish between cyclic shakedown (t<0), cyclic failure (t>0) and cyclic stable (t=0) characters.
4. $k_{0}$ defines the initial value of the new hardening parameter $k_{f}$. It affects the nonlinear behaviour during the cyclic loading; for bigger values, the soil exhibits a more elastic trend at unloading.
5. $k_{1}$ and $k_{2}$ control the cyclic shakedown and the cyclic stable behaviours of soil during load cycles. More specifically the value of $k_{1}$, ranging from 0 to 1, decides whether or not cyclic shakedown will occur; the larger $k_{1}$ is, the greater the chance that the soil will reach the shakedown state. $k_{2}$, on the other hand, defines the degradation rate and normally ranges from 0 to 100.

## 3. Numerical Implementation of the Constitutive Model

The proposed constitutive model is implemented within the user-defined material subroutine UMAT, in ABAQUS [42] enviroment. The main purpose of UMAT is, for each Gauss point, to return the continuum tangent operator (DDSDDE) and update the stresses (STRESS) and solution-dependent state variables (STATEV, such as the projection center (op,oq) and damage factor *d*), based on the incremental strain given by ABAQUS.

### 3.1. Two-Step Euler Integration Method

With the introduction of the distance between the true stress point and the image stress point, it is difficult to analytically derive the continuum tangent operator, as well as the second derivatives of the yield and plastic potential surfaces of the bounding surface model. In this paper, the explicit two-step method is used to integrate the proposed constitutive model.

For a given incremental strain Δε, we have

(26) $$\Delta \sigma^{\left(1\right)}=D_{n}^{ep}:\Delta \varepsilon ;\;\sigma_{n+1}^{\left(1\right)}=\sigma_{n}+\Delta \sigma^{\left(1\right)}=\sigma_{n}+D_{n}^{ep}:\Delta \varepsilon$$

where $\sigma_{n+1}^{\left(1\right)}$ is the first estimate of $\sigma_{n+1}$, based on the given incremental strain, and $D_{n}^{ep}$ denotes the continuum tangent operator at the beginning of the current increment that is obtained via Equation (5). Based on the first estimation $\sigma_{n+1}^{\left(1\right)}$, we can evaluate the continuum tangent operator $D_{n+1}^{ep}$ at the end of the current step and give

(27) $$\begin{array}{c} \Delta \sigma^{\left(2\right)}=\frac{1}{2}\left[D_{n}^{ep}+D_{n+1}^{ep}\right]:\Delta \varepsilon ;\\ \sigma_{n+1}^{\left(2\right)}=\sigma_{n}+\Delta \sigma^{\left(2\right)}=\sigma_{n}+\frac{1}{2}\left[D_{n}^{ep}+D_{n+1}^{ep}\right]:\Delta \varepsilon \end{array}$$

where $\Delta \sigma^{\left(2\right)}$ is the second estimation of the incremental stress using the trapezoidal rule.

Then, the error *E* between the two stress approximations can be expressed as:

(28) $$E=\frac{\sqrt{\left(\sigma_{n+1}^{\left(2\right)}-\sigma_{n+1}^{\left(1\right)}\right):\left(\sigma_{n+1}^{\left(2\right)}-\sigma_{n+1}^{\left(1\right)}\right)}}{\sqrt{\sigma_{n+1}^{\left(2\right)}:\sigma_{n+1}^{\left(2\right)}}}$$

When *E* meets a specified tolerance, the stress state and state variables can be updated and the next step can be taken.

### 3.2. Stress Integration Scheme with Automatic Error Control

The accuracy of the explicit integration scheme is mainly affected by the size of the strain increment Δε. With a small increment size, the explicit integration scheme can achieve higher precision, but loses efficiency.

In order to increase the efficiency of the explicit method, Sloan [43] proposed improving the two-step integration method by subdividing the original incremental size with a pseudo time sub-increment $\Delta T_{i}\left(0<\Delta T_{i}\leq 1\right)$.

The explicit two-step method, with an automated error check, can thus be described mainly as follows (see details in Algorithm 1):

1. The incremental strain Δε is divided, by the pseudo time sub-increment $\Delta T_{i}$, into several sub-increments $\Delta \varepsilon_{i}=\Delta T_{i}\Delta \varepsilon$.
2. Given $\Delta \varepsilon_{i}$, the stress is evaluated according to the Equations (26) and (27) and the error *E*, between the two stress estimates, is calculated using Equation (28). If the error meets a specified tolerance, the stress state and state variables are updated, and it is possible to move on to the next sub-increment. The size of the next sub-increment is then enlarged by a factor.
3. If the error is larger than the tolerance, the current sub-increment result is dropped. The pseudo time $\Delta T_{i}$ has to be decreased to meet the tolerance; its size is adjusted automatically as long as the error control criterion is satisfied.

**Algorithm 1:** Two-Step Explicit Integration Method with Error Control.
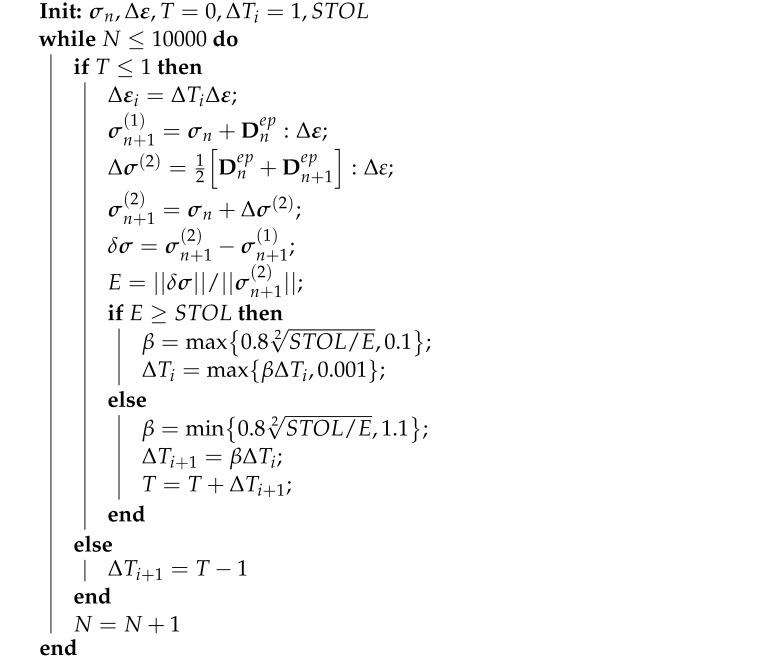


## 4. Experimental–Numerical Comparisons

In this section, the proposed constitutive model implemented in ABAQUS is validated against several undrained cyclic triaxial experiments taken from the literature. Specifically, through the tests conducted on New-field, soft marine and WenZhou clays, the cyclic shakedown, failure and stable behaviours are investigated.

Numerical analyses are performed on a unit cubic element, properly restrained and loaded as described in the following. As boundary conditions, the bottom surface of the cube is fixed in the vertical direction while the two lateral surfaces, which share the origin node with the bottom one, are fixed in the other two directions, respectively. As for the initial and loading conditions, on the other hand, in the first stage, a confinement pressure is applied to the other three unrestrained faces of the cube and, subsequently, a vertical pressure is applied to the top.

The model parameters for the three tests are listed in Table 1.

### 4.1. Cyclic Shakedown Behaviour

Sangrey et al. [44] performed undrained trixial tests to study the cyclic behaviour of Newfield clay. The specimens had an initial void ratio $e_{0}=0.62$; they were firstly consolidated with ${\bar{p}}_{c}^{\prime }=393$ kPa and then loaded by a cyclic load of 180 kPa.

**Figure 10:**
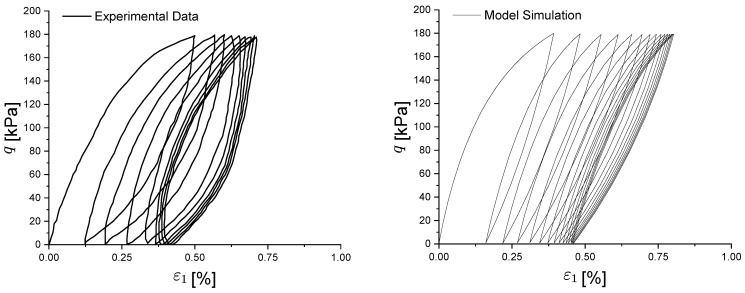
Experimental and numerical comparison of Newfield clay cyclic stress–strain behaviour.

**Figure 11:**
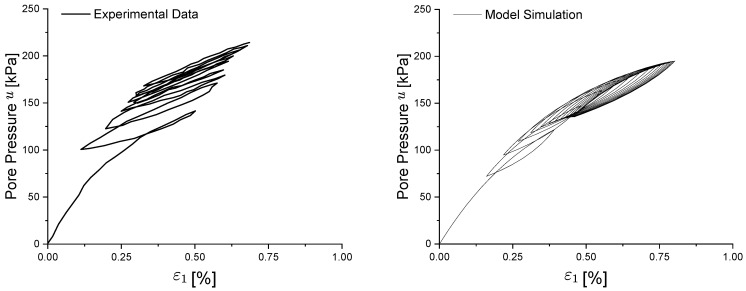
Experimental and numerical comparison of Newfield clay pore pressure–strain evolution under cyclic loading.

**Figure 12:**
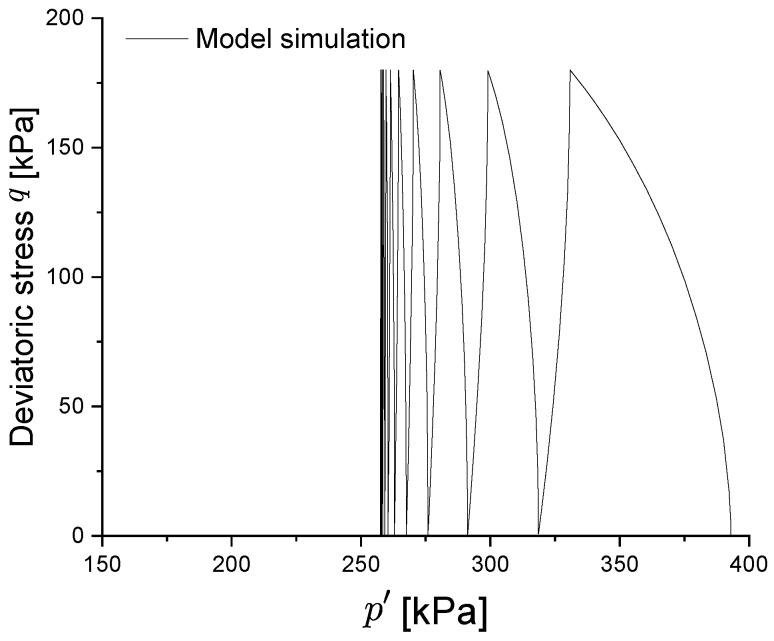
Predicted effective stress path under undrained cyclic loading.

From Figure 10, it can be observed that the closed hysteresis curve of soil under cyclic loading is correctly captured, even if a slightly softer behaviour is modelled; in particular, the phenomenon of shakedown is properly represented after the same number of cyclic loads, at which point the plastic strain level remains constant. The behaviour of the pore pressure (Figure 11) is captured, with a rapid increase during the first three cycles and a tendency to remain stable thereafter. This trend of stabilization can be also observed in the predicted effective stress path of Figure 12.

Therefore, it can be concluded that the proposed model can successfully model the shakedown behaviour when the cyclic load is below the shakedown threshold value.

### 4.2. Cyclic Failure Behaviour

Lei et al. [45] performed a series of cyclic triaxial tests on an ultrasoft type of clay from Binhai, China. The initial conditions of the experiment were ${\bar{p}}_{c}=50$ kPa and $e_{0}=1.297$.

The deformation behaviour of this type of soil is different from others. When the CSR is above a certain threshold value, the soft soil will produce plastic deformation at an increasing rate of each loading cycle, and the soil will tend to fail quickly. Strain at failure is between 0.99 and 1.6%.

**Figure 13:**
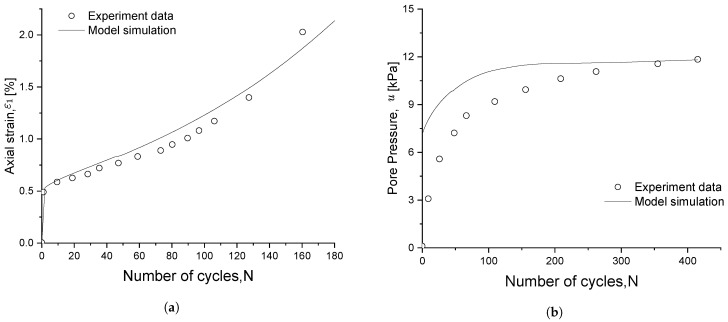
Experimental–numerical comparison of (**a**) axial strain and (**b**) pore pressure evolutions for soft marine clay.

Figure 13a illustrates the experimental–numerical comparison for the axial strain $\varepsilon_{1}$. In detail, it can be appreciated how $\varepsilon_{1}$ increases rapidly after 80 loading cycles; this is also caught by the proposed model, which successfully reproduces the cyclic failure trend. Figure 13b, on the other hand, shows the experimental and numerical results for the pore pressure. It can be appreciated that, in experimentation, this pressure increases rapidly during the first 100 loading cycles and then tends to remain stable. Although what is obtained numerically increases a little bit faster than the experimental data, the trend and the final pore pressure value are realized. This discrepancy is expected because in experiments, not enough time is given for the pore pressure in the soil to balance during the initial phase of cyclic loading. In this case, the accumulation property of pore pressure, especially for long term cycles, is hard to capture and has not been thoroughly studied in the current literature. This difference is also observed in Li’s [47] work on modelling the cyclic behaviour of marine soil.

The cyclic failure behaviour can be evaluated by the degradation index [48] defined by the ratio of secant moduli of the first loading cycle, $G_{S1}$, and the *N*th, $G_{SN}$:

(29) $$\delta =\frac{G_{SN}}{G_{S1}}=\frac{\frac{\sigma_{d}}{\varepsilon_{cN}}}{\frac{\sigma_{d}}{\varepsilon_{c1}}}=\frac{\varepsilon_{c1}}{\varepsilon_{cN}}$$

where $\sigma_{d}$ is the axial cyclic dynamic stress magnitude and $\varepsilon_{C1}$ and $\varepsilon_{CN}$ are the cyclic axial strains at cycles 1 and *N*, respectively.

**Figure 14:**
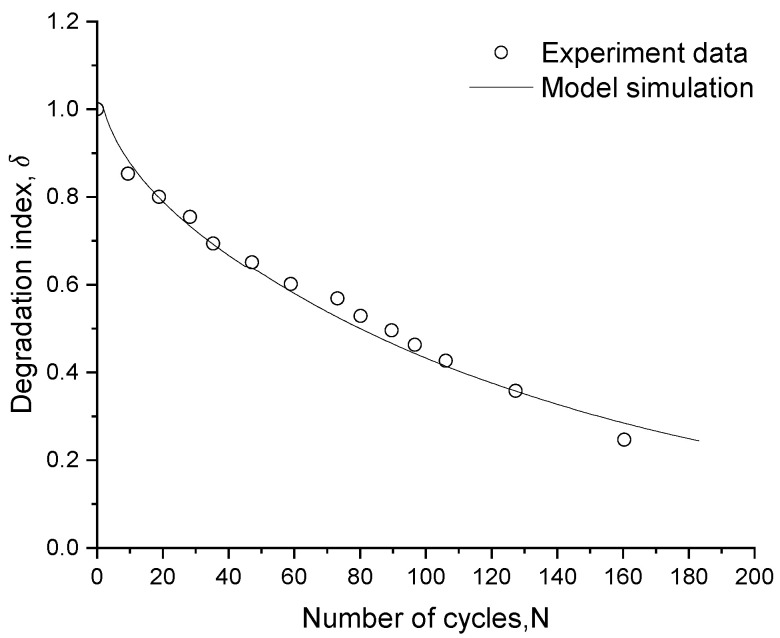
Experimental-numerical comparison of the degradation index evolution for soft marine clay.

From Figure 14, where the numerical–experimental comparison of the degradation index is depicted, a faster decrease in the index during the first 30 cyclic loads can be appreciated; soil stiffness then progressively decreases until it reaches a level that leads to failure. The obtained numerical results match well with the experimentally observed trend; therefore, the proposed damage parameter can efficiently simulate cyclic degradation.

### 4.3. Cyclic Stable Behaviour

Cai et al. [46] performed long-term cyclic triaxial tests on remoulded saturated clay. All the soil samples were firstly consolidated to the same confining pressure ${\bar{p}}_{c}=100$ kPa; then, a series of cyclic loads, with a different CSR from 0.303 to 0.157, were applied vertically to each specimen. Here, experimental data for CSR = 0.208 are chosen for the model validation. The void ratio $e_{0}$ is 1.19

**Figure 15:**
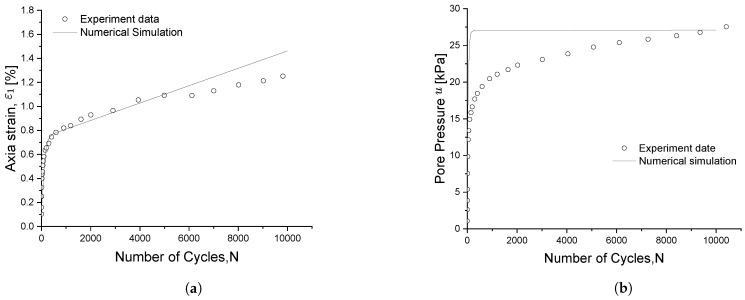
Experimental-numerical comparison of (**a**) axial strain and (**b**) pore pressure evolutions for WenZhou clay.

Figure 15a illustrates the evolution of the axial permanent strain along with the number of loading cycles. Here, it can be appreciated how the strain increases rapidly during the first 1000 cycles and then, for the subsequent cycles, increases at a very slow rate. The soil exhibits a cyclic stable behaviour and the numerical model can simulate it pretty well. Figure 15b, on the other hand, shows the evolution of the pore pressure under long-term cyclic loading. It can be observed that the pore pressure also increases sharply during the first 1000 cycles, since the permanent strain mainly accumulates during the beginning of the cyclic loading and the effective stress reduced faster; even though the numerical results of pore pressure increase faster than those from the experiment, the final pore pressure can be well predicted.

## 5. Conclusions

In the present work a constitutive model based on the bounding surface and stress distance concepts has been developed to properly simulate some important features of cyclic soil behaviour. These include the closed hysteresis curve, degradation and most importantly the three types of cyclic behaviour under various CSRs, i.e., shakedown, stable and failure.

Unlike other bounding surface models, the proposed model adopts a new uniform interpolation function of the plastic modulus for the whole loading process. In addition a new damage factor, along with the deformation type parameter, is incorporated into the hardening modulus, which allows it to vary flexibly with the accumulation of plastic shear strain and thus achieve different cyclic deformation behaviours. The model also adopts the concept of a “vanishing elastic domain”; hence, it does not require complex kinematic hardening rules, and it only needs to record the stress reversal points, thus enabling the modelling of soil hysteretic cycles.

Comparisons between the obtained numerical results and the experimental ones from literature have been conducted, validating the proposed model and confirming its ability to correctly capture and reproduce key aspects of the cyclic behaviour of clays.

Future work will be devoted to extending the model to account for anisotropy, as well as gaining a deeper understanding of the fluid–mechanical interaction.

## Figures and Tables

**Table 1:** Model parameters values.

Parameters	Newfield Clay	Soft Clay	WenZhou Clay
	Sangrey et al. [44]	Lei et al. [45]	Cai et al. [46]
μ	0.17	0.3	0.3
λ	0.051	0.065	0.055
κ	0.011	0.035	0.025
$M_{cs}$	0.83	0.57	0.85
*r*	1.6	1.17	1.43
*n*	1.8	2	2
*t*	−1	1	-
$k_{0}^{un}$	5	19	20.5
$k_{0}^{re}$	7	24	26
$k_{1}^{un}$	0.13	-	0.355
$k_{1}^{re}$	0.4	0.7	-
$k_{2}^{un}$	20	-	18
$k_{2}^{re}$	20	15	-

Note: The superscripts “re” and “un” of *k*_0_, *k*_1_ and *k*_2_ indicate reloading and unloading stage, respectively; ‘-’ stands for null value.

## Data Availability

Not applicable.

## Nomenclature

$D^{e},D^{ep}$	Elasticity tensor and elasto-plastic tangent operator.
*E*	Relative error norm of two estimated stresses.
$e,e_{cs}$	Void ratio and critical void ratio.
F,g	Bounding surface and plastic potential surface.
$G_{S1},G_{SN}$	Secant moduli at loading cycles 1 and *N*.
$h_{b},h_{f}$	Plastic modulus at image stress and interpolation plastic modulus.
*h*	Plastic modulus at true stress.
$k_{0},k_{1},k_{2}$	Cyclic plasticity parameters.
K,G	Bulk modulus and shear modulus.
$k_{f},d$	Hardening parameter and damage parameter.
*L*	Loading index.
$M_{cs}$	Critical stress ratio.
$m_{p}$	Length of the normal vector to plastic potential.
n,r	Bounding surface shape parameters.
$\tilde{n},m$	Normal vectors to the bounding surface and plastic potential.
(op,oq)	Projection centre coordinates.
$p_{c}^{\prime },{\bar{p}}_{c}$	Preconsolidation pressure and hardening internal variable of bounding surface.
${\bar{p}}^{\prime },\bar{q}$	Image stress on the bounding surface.
$p^{\prime },q$	Mean effective stress and deviatoric stress.
*t*	Parameter used to distinguish cyclic shakedown and failure.
*T*	Pseudo time.
*v*	Specific volume.
β	Scaling factor of sub-increment size.
γ	Distance ratio.
$\Gamma_{o}$	Intersection point of the CSL and the void ratio axis at $p^{\prime }=1$.
$\varepsilon_{v},\varepsilon_{s}$	Volumetric strain and shear strain
$\varepsilon_{d}^{p},\varepsilon_{v}^{p}$	Plastic deviatoric strain and plastic volumetric strain.
$\varepsilon_{c1},\varepsilon_{cn}$	Cyclic axial strains at cycles 1 and *N*.
$\varepsilon_{1},\varepsilon_{3}$	Axial strain and lateral strain.
$\varepsilon ,\varepsilon^{e},\varepsilon^{p}$	Total strain tensor, elastic strain tensor and plastic strain tensor.
κ	Elastic slope in $e-\ln p^{\prime }$ space.
λ	Slope of the normal consolidation line.
Λ	Plastic multiplier.
μ	Poisson’s ratio.
$\bar{\rho }$	Euclidean distance between image stress and projection centre.
ρ	Euclidean distance between image stress and true stress.
$\sigma_{1}^{\prime },\sigma_{3}^{\prime }$	Axial and lateral effective stresses.
$\sigma_{d}$	Magnitude of axial cyclic stress.
$\sigma^{\prime },{\bar{\sigma }}^{\prime }$	Effective stress tensor and the corresponding stress on bounding surface.
ϕ	Drained friction angle.
